# 

**Published:** 1913-04-05

**Authors:** 


					js The Hospr^vL4Xx<^^<?/T^U April 5, 1913. J
L special hit Hi Jp ^
(. SPECIAL Jpg, NUMBER.
0
HOS
The Modern Newspaper of Administrative
Medicine and Institutional Life.
Telegraphic Address ; OSPEDALE. RAND, LONDON. Telephone Number: 2734 GERRARD.
No. 1396, Vol. L1V. Saturday,
April 5th, 1913.
PRICE ONE PENNY

				

